# Dual inhibition of Fes and Flt3 tyrosine kinases potently inhibits Flt3-ITD^+^ AML cell growth

**DOI:** 10.1371/journal.pone.0181178

**Published:** 2017-07-20

**Authors:** Mark C. Weir, Sabine Hellwig, Li Tan, Yao Liu, Nathanael S. Gray, Thomas E. Smithgall

**Affiliations:** 1 Department of Microbiology and Molecular Genetics, University of Pittsburgh School of Medicine, Pittsburgh, Pennsylvania, United States of America; 2 Department of Biological Chemistry and Molecular Pharmacology, Harvard Medical School and Department of Cancer Biology, Dana-Farber Cancer Institute, Boston, Massachusetts, United States of America; Pennsylvania State University, UNITED STATES

## Abstract

Acute myelogenous leukemia (AML) is often associated with activating mutations in the receptor tyrosine kinase, Flt3, including internal tandem duplications (ITDs) within the regulatory juxtamembrane region. Previous studies have linked Flt3-ITD to the activation of the Fes protein tyrosine kinase in AML, and RNAi-knockdown studies suggest that Fes may be required for Flt3 function. In this study, we tested Fes inhibitors from three different chemical classes for their growth-suppressive activity against Flt3-ITD^+^ myeloid leukemia cell lines (MV4-11, MOLM-13 and MOLM-14) vs. myeloid cells with wild-type Flt3 (THP-1). All Fes inhibitors selectively inhibited the growth of Flt3-ITD^+^ AML cells, with IC_50_ values for diaminopyrimidine and pyrrolopyridine inhibitors ranging from 19 to 166 nM. In contrast, a pyrazolopyrimidine inhibitor was less potent in Flt3-ITD^+^ AML cells, with IC_50_ values in the 1.0 μM range. In vitro kinase assays showed that the most potent inhibitors of Flt3-ITD^+^ AML cell proliferation blocked both Fes and Flt3-ITD kinase activity, while the pyrazolopyrimidine was more selective for Fes vs. Flt3-ITD. All three inhibitors induced significant apoptosis in Flt3-ITD^+^ AML cells, with potency equivalent to or greater than the established Flt3-ITD inhibitor, tandutinib. Transformation of TF-1 cells with Flt3-ITD resulted in constitutive activation of endogenous Fes, and rendered the cells highly sensitive to all three Fes inhibitors with IC_50_ values in the 30–500 nM range. The pyrrolopyridine compound also induced apoptotic responses in patient-derived Flt3-ITD^+^ AML bone marrow cells but not in normal bone marrow mononuclear cells. These results demonstrate that Fes kinase activity contributes to Flt3-ITD signaling in AML, and suggests that dual inhibition of both Flt3 and Fes may provide a therapeutic advantage for the treatment of Flt3-ITD^+^ AML.

## Introduction

Acute myelogenous leukemia (AML) is the most common hematologic malignancy in adults [1]. The current standard of care for AML typically involves cytotoxic chemotherapy, which has changed little in the last 40 years and has resulted in a stagnant overall survival rate of approximately 25% [2,3]. While numerous cytogenetic abnormalities and mutations have been identified in AML, the receptor tyrosine kinase FMS-like tyrosine kinase 3 (Flt3) is mutated in approximately 30% of all AML cases [4,5]. Flt3 mutations occur as internal tandem duplications (ITDs), in-frame duplications of varying length within the juxtamembrane region, or as point mutations, most commonly at position D835 within the activation loop of the kinase domain [6,7]. Both types of mutations result in a constitutively active kinase that drives AML pathogenesis. Flt3-ITD mutations in particular are associated with a poor prognosis relative to other forms of AML [8,9].

Fes belongs to a unique family of non-receptor tyrosine kinases and is expressed in hematopoietic cells, particularly in the myeloid lineage [10,11]. Originally identified as the cellular homolog of the transforming oncogene present in several avian and feline sarcoma viruses, Fes kinase activity is tightly regulated in cells [12]. Fes normally functions as a signaling mediator downstream of growth factor, cytokine and immune cell receptors and is involved in hematopoietic cell growth, survival and differentiation as well as innate immune responses [13].

Previous work by Voisset and colleagues has implicated Fes as an important downstream signaling partner for Flt3-ITD in AML [14]. They discovered that Fes was expressed and constitutively active in two Flt3-ITD^+^ AML cell lines, MV4-11 and MOLM-14, as well as in primary AML bone marrow samples. Knockdown of Fes expression in both cell lines decreased cell growth to a similar extent as knockdown of Flt3-ITD itself. Furthermore, the activity of Flt3-ITD downstream signaling mediators, particularly STAT5 and PI3K, were also substantially decreased in Fes-knockdown cells. Co-immunoprecipitation studies demonstrated that the two kinases physically interact, and knockdown of Flt3-ITD led to a decrease in Fes kinase activity, supporting the idea that Fes is a downstream mediator of Flt3-ITD oncogenic signaling [14]. Finally, treatment of primary AML patient samples with the Flt3 inhibitor, SU5416, reduced both Flt3 and Fes activation. These data strongly suggest that Fes is essential for the activation of signaling pathways downstream of Flt3-ITD, and that inhibition of Fes kinase activity may be therapeutically beneficial in AML. In the present study, we explored the role of Fes kinase activity in AML cell growth using a panel of ATP-site inhibitors selective for Fes, selective for Flt3, or with dual activity for Fes and Flt3. Our results show that while inhibition of Fes kinase activity alone is sufficient to block AML cell growth, inhibitors with dual activity against both Flt3-ITD and Fes are even more active, with IC_50_ values in the low nM range in multiple Flt3-ITD^+^ AML cell lines.

## Materials and methods

### Cell culture, reagents, and antibodies

The human AML cell lines MV4-11 (CRL-9591) and THP-1 (TIB-202) were obtained from the American Type Culture Collection (ATCC), while the AML cell lines MOLM-13 (ACC-544) and MOLM-14 (ACC-777) were obtained from the Leibniz-Institute DSMZ-German Collection. MV4-11 and THP-1 cells were cultured in RPMI 1640 medium supplemented with 10% fetal bovine serum (FBS; Gemini BioProducts), 2 mM L-glutamine, 100 units/ml of penicillin, 100 μg/ml of streptomycin sulfate, and 0.25 μg/ml of amphotericin B (Antibiotic-Antimycotic; Gibco/ThermoFisher). MOLM-13 and MOLM-14 cells were cultured in RPMI 1640 medium supplemented with 20% FBS and Antibiotic-Antimycotic. TF-1 cells (CRL-2003) were obtained from the ATCC and were cultured in RPMI-1640 medium supplemented with 10% FBS, Antibiotic-Antimycotic, and 1 ng/mL of recombinant human GM-CSF (ThermoFisher). Human 293T cells (CRL-3216) and Hs27 feeder fibroblasts (CRL-1634) were obtained from the ATCC and cultured in Dulbecco's modified Eagle's medium (DMEM) containing 10% FBS and Antibiotic-Antimycotic. Culture media and supplements were obtained from Life Technologies except where noted.

Primary antibodies used in this study were obtained from Santa Cruz Biotechnology (pY99 anti-phosphotyrosine, SC-7020; Fes, N-19) and Cell Signaling Technologies (Flt3, 3462S). The Fes activation loop phosphospecific antibody (pY713) was previously developed by our group [15]. Alkaline phosphatase-linked secondary antibodies were purchased from Southern Biotech. Tandutinib and staurosporine were purchased from LC Laboratories and reconstituted as 10 mM stock solutions in DMSO.

### Growth inhibition and apoptosis assays

Cells were seeded at a density of 100,000 cells per 1.0 ml in 48-well culture plates. Compounds were added to various concentrations while holding the DMSO concentration constant at 0.1%. Cell viability was assessed 72 hours later using the CellTiter-Blue Cell Viability assay (Promega) according to the manufacturer’s protocol and a SpectraMax Multi-Mode Microplate Reader (Molecular Devices). IC_50_ values were determined by non-linear regression analysis of concentration-response curves using the GraphPad PRISM software package (version 6.0). Apoptosis was determined using the same cell culture conditions and the Apo-ONE Homogeneous Caspase-3/7 Assay (Promega) and the manufacturer’s protocol.

### Immunoprecipitation

AML cells (2.5 x 10^6^ in 5 mL) were cultured in the presence of kinase inhibitors or DMSO alone for 6 to 16 hours prior to lysis by sonication in RIPA buffer (50 mM Tris-HCl, pH 7.4, 150 mM NaCl, 1 mM EDTA, 1% Triton X-100, 0.1% SDS, 1% sodium deoxycholate) supplemented with 1 mM sodium orthovanadate, 1 mM sodium fluoride, 5 units/ml Benzonase (Novagen), and SigmaFAST EDTA-free protease inhibitor cocktail (Sigma). Flt3 and Fes were immunoprecipitated from 1 mg of cell lysate with 2 μg of antibody and 25 μL of protein G-Sepharose beads (Life Technologies) overnight at 4°C. Immunoprecipitates were washed three times by resuspension in 1.0 ml of RIPA buffer. Immunoprecipitated proteins were separated by SDS-PAGE, transferred to PVDF membranes and probed with the indicated antibodies followed by alkaline phosphatase-linked secondary antibodies. CDP-Star Western Blot Chemiluminescence Reagent (Perkin-Elmer) was used for detection.

### In vitro kinase assays

Recombinant protein kinase assays were performed using the FRET-based Z’-LYTE Kinase Assay according to the manufacturer’s instructions (Thermo Fisher Scientific). Recombinant Flt3-WT, Flt3-ITD, and Flt3-D835Y kinase domain proteins were purchased from Life Technologies, while recombinant Fes was expressed in *E*. *coli* and purified as described previously [16]. The Tyr-2 peptide was used as substrate for all recombinant kinases. Kinases were preincubated with inhibitors for 30 minutes, followed by addition of ATP and peptide substrate for 1 hour. Reactions were quenched by addition of development reagent, followed by incubation for an additional hour prior to fluorescence measurements on a SpectraMax M5 microplate reader. IC_50_ values were calculated by non-linear regression analysis of the resulting concentration-response curves using GraphPad PRISM.

### Retrovirus production and cellular transduction

Full-length cDNA clones for human wild-type FLT3, FLT3-ITD (based on the sequence of the insertion found in MV4-11 AML cells) and the FLT3-D835Y were subcloned into the retroviral expression vector pMSCVneo (Clontech). To prepare recombinant retroviruses, 293T cells were plated at a density of 1.5 x 10^6^ cells per 100 mm dish one day prior to transfection. Each retroviral expression vector (15 μg) was combined with an amphotropic packaging plasmid (15 μg) in 450 μL of water to which 60 μL of 2 M calcium chloride was added. This solution was mixed with 500 μL of HEPES-buffered saline (50 mM HEPES, 1.5 mM Na_2_HPO_4_, 280 mM NaCl, pH 7.4) and incubated on ice for 10 minutes. The solution was then added dropwise to the 293T cells in fresh culture medium. After overnight incubation, the medium was replaced with 6 mL of fresh medium, and the viral supernatant was collected 48 hours later. All retroviral supernatants were filtered through a 0.22 μM syringe filter, and stored at -80°C.

For each retroviral transduction, TF-1 cells (1 x 10^6^) were suspended in 5 mL of undiluted viral supernatant and plated in one well of a 6-well tissue culture dish. Polybrene (Sigma) was added to a final concentration of 4 μg/mL and the TF-1 cells were centrifuged at 1000 x g for 3 hours to enhance retroviral mediated gene transfer. After centrifugation, the virus was removed and replaced with fresh medium. Two days later, G-418 was added to 800 μg/mL to select the transduced cell population; following selection, cells were maintained in G-418 at 400 μg/mL.

### Culture and analysis of apoptosis in primary AML bone marrow samples

Hs27 feeder fibroblasts were mitotically inactivated by treating a confluent monolayer of cells in a 10 cm tissue culture dish with 10 μg/mL of mitomycin C (MMC; Sigma) for 6 hours and cryopreserved until use. De-identified primary human AML bone marrow samples were obtained from the Health Sciences Tissue Bank at the University of Pittsburgh as cryopreserved specimens. The Flt3-ITD genotype of each primary AML bone marrow sample was confirmed by PCR and nucleotide sequence analysis of *FLT3* exons 14–15, where ITD mutations commonly occur (data not shown). Cryopreserved bone marrow mononuclear cells were purchased from Lonza (2S-101D) for evaluation of non-specific cytotoxicity. All primary cells were cultured using the method of Klco [17] as follows: MMC-treated Hs27 cells (5 x 10^5^) were plated in each well of a 6-well plate and allowed to attach overnight. Primary AML samples were thawed quickly, mixed with in 10 mL of PBS, centrifuged at 1000 x g for 3 min and then resuspended in Iscove’s modified Dulbecco’s medium supplemented with 15% FBS, antibiotic/antimycotic, 2 mM L-glutamine, and human SCF (50 ng/mL), IL-3 (10 ng/mL), and IL-6 (20 ng/mL; all cytokines from Peprotech). The primary AML bone marrow samples were then gently plated on the MMC-treated Hs27 fibroblast feeder layers. Primary AML bone marrow cells were transferred to fresh feeder layers in 48-well plates one day later, and test compounds were added followed by incubation for an additional 48 to 72 hours. Cells were fixed and stained with an antibody to the early apoptotic marker, phosphatidylserine (Millipore; 16–256) according to the manufacturer’s instructions. Cells were then analyzed on a BD Accuri C6 Flow cytometer and the resulting data were evaluated using the BD CSampler software.

## Results

### Fes inhibitors induce growth arrest and apoptosis in Flt3-ITD^+^ AML cells

**Fig 1:**
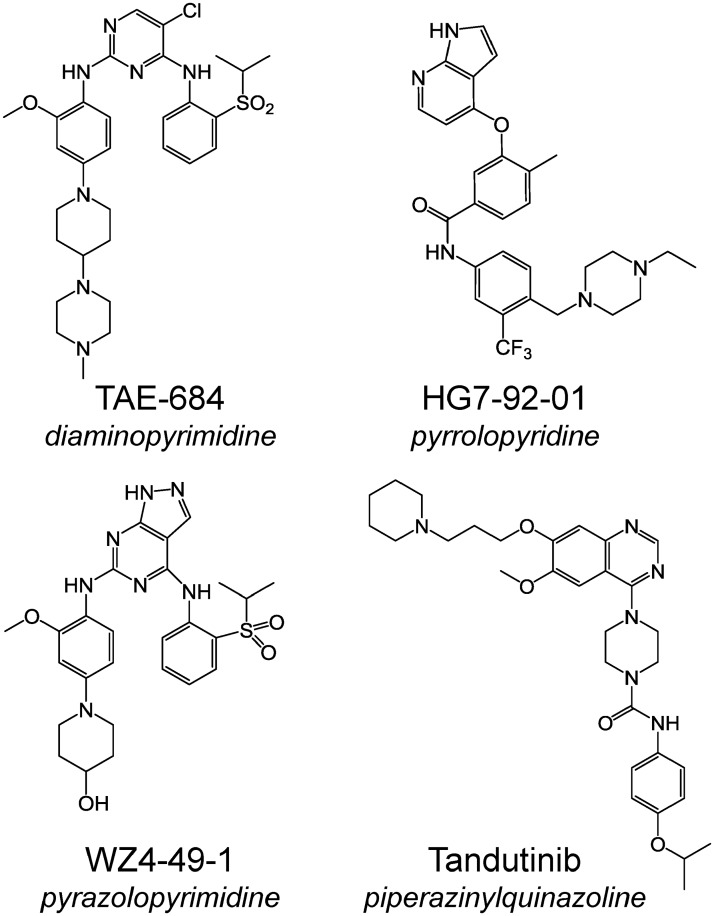
Chemical structures of the tyrosine kinase inhibitors used in this study. Compounds include the diaminopyrimidine TAE-684, the pyrrolopyridine HG7-92-01, and the pyrazolo-pyrimidine, WZ4-49-1. The discovery and characterization of these compounds as cell-active inhibitors of Fes kinase activity is described in Hellwig, et al. [16]. Tandutinib (CT-53518; MLN-518) is a piperazinyl quinazoline inhibitor of Flt3-ITD, c-Kit and the PDGF-R [18,19] that does not inhibit Fes in vitro or in cells (this study).

**Fig 2:**
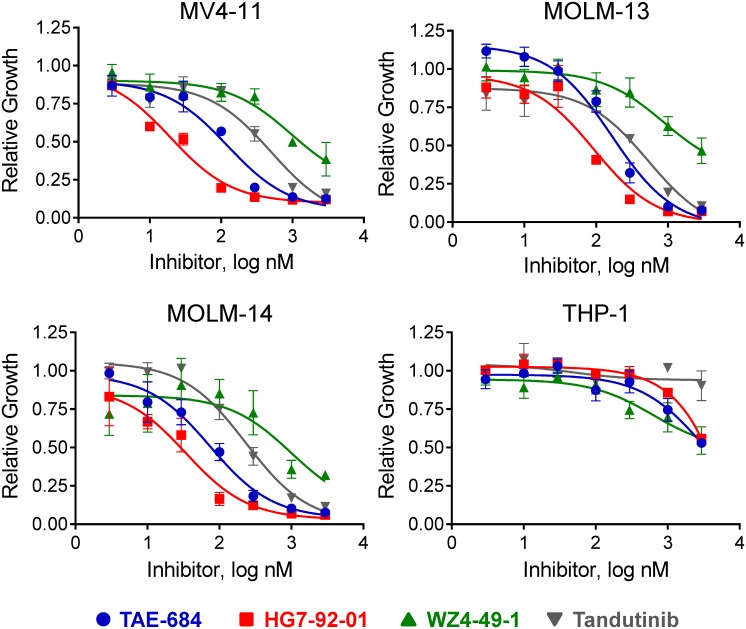
Fes tyrosine kinase inhibitors induce growth arrest in Flt3-ITD^+^ AML cell lines. A) The Flt3-ITD^+^ cell lines MV4-11, MOLM-13, and MOLM-14, along with THP-1 (wild-type *FLT3*) were incubated with each of the kinase inhibitors shown over a range of concentrations, and cell viability was measured 72 h later using the CellTiter-Blue Assay (see Materials and methods). Raw fluorescence data were corrected for background and then normalized to cultures grown in the presence of the DMSO carrier solvent. Each assay condition was performed in triplicate, and results are presented as the mean of the normalized values ± the SD. The resulting dose-response curves were fitted by non-linear regression analysis, and the resulting IC_50_ values are shown in Table 1.

To test the hypothesis that inhibition of Fes kinase activity may be of therapeutic value in AML, we first investigated the effects of Fes kinase inhibitors [16] from three distinct chemical classes (Fig 1) on the growth of AML cell lines either wild-type for Flt3 (THP-1) or expressing Flt3-ITD (MV4-11, MOLM-13 and MOLM-14). All of the Fes kinase inhibitors tested selectively blocked the growth of the Flt3-ITD^+^ AML cell lines (Fig 2). Inhibitors from the diaminopyrimidine (TAE-684) and pyrrolopyridine (HG7-92-01) classes were the most potent, with IC_50_ values ranging from 19 to 166 nM (Table 1). The pyrazolopyrimidine inhibitor, WZ4-49-1, was less potent, yielding IC_50_ values of close to 1.0 μM in all three Flt3-ITD^+^ AML cell lines (Fig 2 and Table 1). Notably, both TAE-684 and HG7-92-01 were more potent than the previously reported Flt3 inhibitor, tandutinib [18,19], which yielded IC_50_ values ranging from 232 to 502 nM in the Flt3-ITD^+^ AML cell lines. None of these compounds had significant growth inhibitory activity against THP-1 cells, which are wild-type for FLT3 and express Fes [20], but are transformed by tyrosine kinase-independent mechanisms that may involve an *MLL-AF9* translocation [21,22] as well as a point mutation in the *NRAS* locus [23].

**Table 1:** In vitro inhibitor specificity profiles with recombinant Fes and Flt3 kinases. In vitro kinase assays were performed with recombinant full-length Fes as well as wild-type (WT), ITD and D835Y forms of the Flt3 kinase domain (Z’-Lyte assay; see Materials and methods). Each inhibitor was tested in triplicate over a range of concentrations with the ATP concentration set to the K_m_ value for each kinase. IC_50_ values were calculated by non-linear regression analysis of the resulting dose-response curves. Also included in the table are the IC_50_ values for growth suppression by each compound in four AML cell lines, calculated from the dose-response curves shown in Fig 2. Note that MV4-11, MOLM-13, and MOLM-14 are Flt3-ITD^+^, while THP-1 is wild-type for Flt3.

	Growth Suppression in AML Cell Lines (IC_50_, nM)				In vitro Kinase Assay (IC_50_, nM)			
**Compound**	MV4-11	MOLM-13	MOLM-14	THP-1	Fes	Flt3-WT	Flt3-ITD	Flt3-D835Y
**TAE-684**	123	166	74	> 3,000	569	536	120	218
**HG7-92-01**	19	93	34	> 3,000	527	536	149	3,646
**WZ4-49-1**	965	884	994	> 3,000	494	3,138	1,390	642
**Tandutinib**	502	532	232	> 3,000	>100,000	580	788	7,370

**Fig 3:**
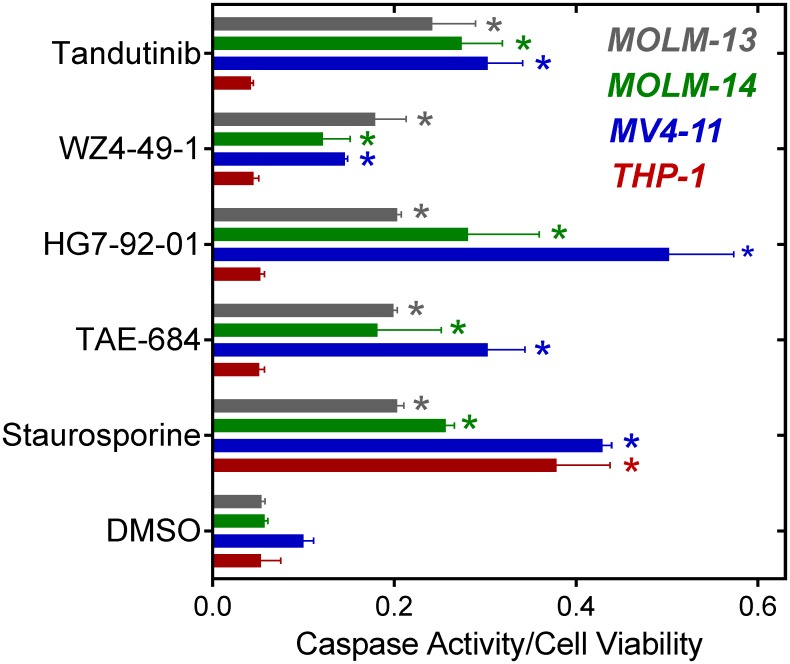
Fes tyrosine kinase inhibitors induce apoptosis in Flt3-ITD^+^ AML cell lines. The Flt3-ITD^+^ cell lines MV4-11, MOLM-13, and MOLM-14, along with THP-1 (wild-type *FLT3*), were incubated with each of kinase inhibitors shown at a final concentration of 1 μM and apoptosis was measured 72 h later using the Apo-ONE Caspase 3/7 Assay (Promega). Cell viability was measured in parallel using the CellTiter-Blue assay, and the resulting data are presented as the ratio of Caspase activity to cell viability as per the manufacturer’s protocol. All assays were performed in triplicate, and data are presented as the mean ratios ± SD. Asterisks indicate statistical significance between each treatment group vs. the corresponding DMSO control group with p < 0.05 (Student’s t-test).

Next, we investigated whether Fes inhibitors were able to induce programmed cell death in Flt3-ITD^+^ AML cells. MV4-11, MOLM-13, MOLM-14 and THP-1 cells were treated with each inhibitor at a concentration of 1.0 μM, and apoptosis was assayed as Caspase 3/7 activity three days later. The three Fes inhibitors as well as tandutinib each induced significant apoptosis under these conditions in all three Flt3-ITD^+^ AML cell lines but not in THP-1 cells, consistent with the observation that Fes is constitutively active in Flt3-ITD^+^ AML cells [14] (Fig 3). Both TAE-684 and HG7-92-01 induced a percentage of apoptotic cells equal to or greater than that observed with tandutinib, while the effect of WZ4-49-1 was less pronounced. As a positive control, all four cell lines were also treated with the broad-spectrum kinase inhibitor staurosporine, which induced a strong apoptotic response in each case as expected.

### In vitro kinase assays reveal dual inhibitor specificity for Fes and Flt3

Data presented so far show that Fes inhibitors were able to induce growth arrest and apoptosis in Flt3-ITD^+^ AML cells. However, the differences in potency across the compounds suggested that inhibition of additional kinase targets, including Flt3-ITD itself, may account for the higher potency of TAE-684 and HG7-92-01 compared to the pyrazolopyrimidine, WZ4-49-1. Previous KINOMEscan analyses of the kinome-wide target specificity profiles of these compounds support this idea [16]. These KINOMEscan data show that the more potent compounds, TAE-684 and HG7-92-01, bind to a wider range of kinases (including Flt3) compared to WZ4-49-8, a close structural homolog of WZ4-49-1 that is more selective for Fes and does not bind to Flt3. That said, the KINOMEscan target profiling approach involves an indirect binding assay [24], and while very useful for kinome-wide analysis of inhibitor target specificity, does not provide direct evidence of inhibitory potency. Therefore, to compare the inhibitory profiles of each compound for Fes and Flt3 directly, we performed in vitro kinase assays using recombinant purified full-length Fes and three Flt3 kinase domains (wild-type plus the AML-associated mutants, Flt3-ITD and Flt3-D835Y). As shown in Table 1, the most potent inhibitors of Flt3-ITD^+^ AML cell growth and survival inhibited both Fes and Flt3 with similar potencies. For example, the pyrrolopyridine compound HG7-92-01 inhibited both Fes and wild-type Flt3 in the 500 nM range, and also inhibited the Flt3-ITD kinase domain with an IC_50_ value of 149 nM. A remarkably similar inhibitory profile was also observed for TAE-684 with Fes, wild-type Flt3, and Flt3-ITD. In contrast, the pyrazolopyrimidine compound WZ4-49-1 showed a similar potency for Fes as the other two compounds (494 nM), but was less potent against the kinase domains of wild-type Flt3 (3,138 nM) and Flt3-ITD (1,390 nM). We also investigated the inhibitory profile for tandutinib using the in vitro kinase activity, and found that while it is active against the wild-type and ITD forms of Flt3 (IC_50_ values of 580 and 788 nM, respectively), it had no effect on Fes kinase activity in vitro (highest concentration tested was 100 μM). Tandutinib inhibited Flt3-ITD^+^ AML cell proliferation with intermediate potency compared to the other compounds, which may be due in part to its lack of activity against Fes (Table 1).

**Fig 4:**
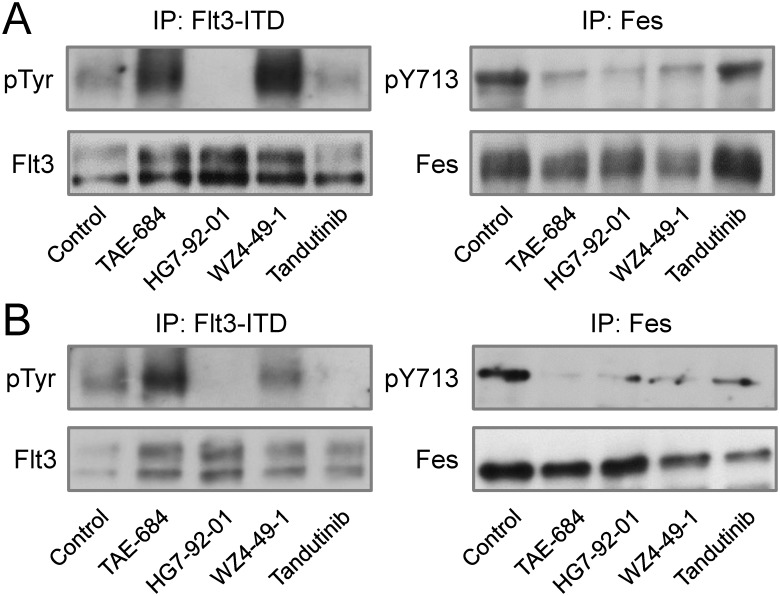
Immunoblot analysis of Flt3-ITD and Fes tyrosine phosphorylation following inhibitor treatment of Flt3-ITD^+^ AML cells. MV4-11 (A) and MOLM-14 (B) cells were treated with TAE-684, HG7-92-01, and WZ4-49-1 at their calculated in vitro IC_90_ values for inhibition of Fes for 16 hours (or Flt3-ITD in the case of tandutinib for 6 hours). Fes and Flt3 kinase proteins were then immunoprecipitated from cell lysates, followed by immunoblotting with antibodies to phosphotyrosine (pTyr), the Fes activation loop phosphotyrosine (pY713), as well as the Flt3 and Fes proteins. This experiment was performed in triplicate with comparable results, and a representative example is shown.

To determine whether a similar specificity profile was observed in Flt3-ITD^+^ AML cells, we treated MV4-11 and MOLM-14 cultures with each compound at its calculated IC_90_ value for Fes from the in vitro kinase assays (or Flt3-ITD in the case of tandutinib). Flt3 and Fes were then immunoprecipitated from cell lysates, and immunoblotted for overall Flt3 phosphotyrosine content or for activation loop phosphorylation in the case of Fes. As shown in Fig 4, HG7-92-01 inhibited Flt3-ITD and Fes in both cell lines, while WZ4-49-1 only inhibited Fes, consistent with the in vitro kinase assay results. On the other hand, TAE-684 had no effect on cellular Flt3-ITD phosphotyrosine content but did inhibit Fes. Previous studies have also reported a similar difference between in vitro and cellular assays with TAE-684 (see Discussion). Treatment of cells with tandutinib at the in vitro IC_90_ value for Flt3-ITD reduced Flt3-ITD phosphotyrosine content in both Flt3-ITD^+^ AML cell lines but had little effect on Fes, consistent with the in vitro kinase assay.

### Transformation of human myeloid cells with Flt3-ITD but not Flt3-D835Y activates endogenous Fes and induces sensitivity to Fes inhibitors

**Fig 5:**
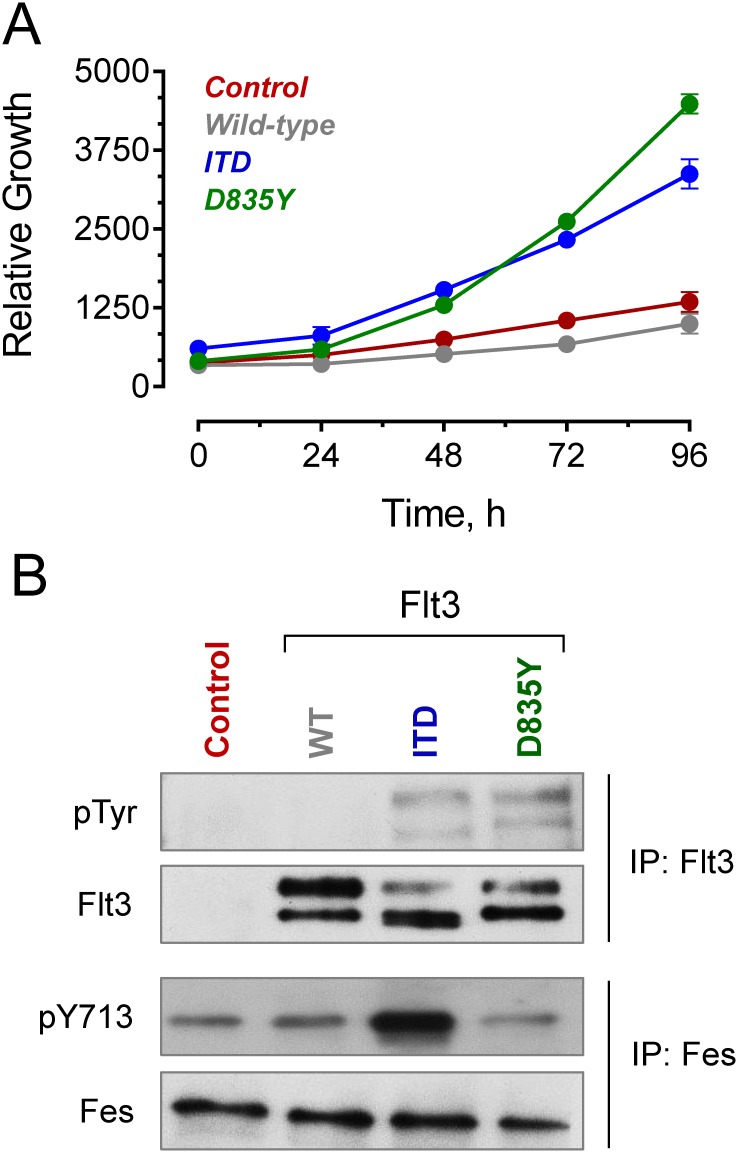
Active mutants of Flt3 (ITD and D835Y) transform human TF-1 cells to cytokine independent growth. A) The human myeloid cell line TF-1 was transduced with recombinant retroviruses carrying wild-type (WT), ITD, or D835Y forms of Flt3, or with an empty vector as control. Following selection with G418, each of the TF-1 cell populations was cultured in the absence of GM-CSF, and viable cell outgrowth was monitored daily by CellTiter-Blue assay for 4 days. Each population was assayed in triplicate, and results are presented as the mean value ± the SD. B) Fes and Flt3 kinase proteins were immunoprecipitated from each of the cell populations shown in part A, followed by immunoblotting with antibodies to phosphotyrosine (pTyr), the Fes activation loop phosphotyrosine (pY713), as well as the Flt3 and Fes proteins. This experiment was performed in triplicate with comparable results, and a representative example is shown.

We next investigated whether transformation with Flt3-ITD is directly responsible for activation of Fes in a myeloid cell background. For these studies, we used the human myeloid leukemia cell line TF-1, which expresses endogenous Fes but not Flt3. These cells are dependent on the hematopoietic cytokine GM-CSF for growth and survival, and have been previously shown to undergo transformation to a cytokine-independent phenotype following introduction of Bcr-Abl, the oncogenic tyrosine kinase responsible for CML [25]. To determine if TF-1 cells are also susceptible to transformation by active mutants of Flt3 associated with AML, we transduced TF-1 cells with retroviral vectors for wild-type Flt3, a Flt3-ITD mutant identical to the one found in MV4-11 cells, and an active Flt3 point mutant, D835Y. As shown in Fig 5A, TF-1 cells transduced with the ITD and D835Y mutants of Flt3 underwent transformation to cytokine-independent growth, while cells expressing wild-type Flt3 did not.

We next explored the expression and activity of both Flt3 and Fes kinases in control and Flt3-transformed TF-1 cell populations. Each kinase protein was immunoprecipitated, and then assessed for activity by immunoblotting with antibodies for the Fes activation loop phosphotyrosine (pTyr713) or with an anti-phosphotyrosine monoclonal antibody (pY99) in the case of Flt3 as described above for MV4-11 cells. As shown in Fig 5B, the Flt3 kinase protein was readily detected in the three TF-1 cell populations transduced with Flt3 retroviruses, but was absent in the parental TF-1 cells as expected. However, only the ITD and D835Y forms of Flt3 showed reactivity with the anti-phosphotyrosine antibody, indicating that they are constitutively active. These findings are consistent with the GM-CSF-independent outgrowth of these two cell populations compared to those expressing wild-type Flt3 or the vector control cells. Endogenous Fes protein was also readily detected in all four cell populations. Interestingly, endogenous Fes was active only in TF-1 cells transformed with Flt3-ITD, and not in the Flt3-D835Y cells or either of the control cell populations. This observation suggests important differences between these two active forms of Flt3 in terms of downstream effector kinase coupling (see Discussion).

**Fig 6:**
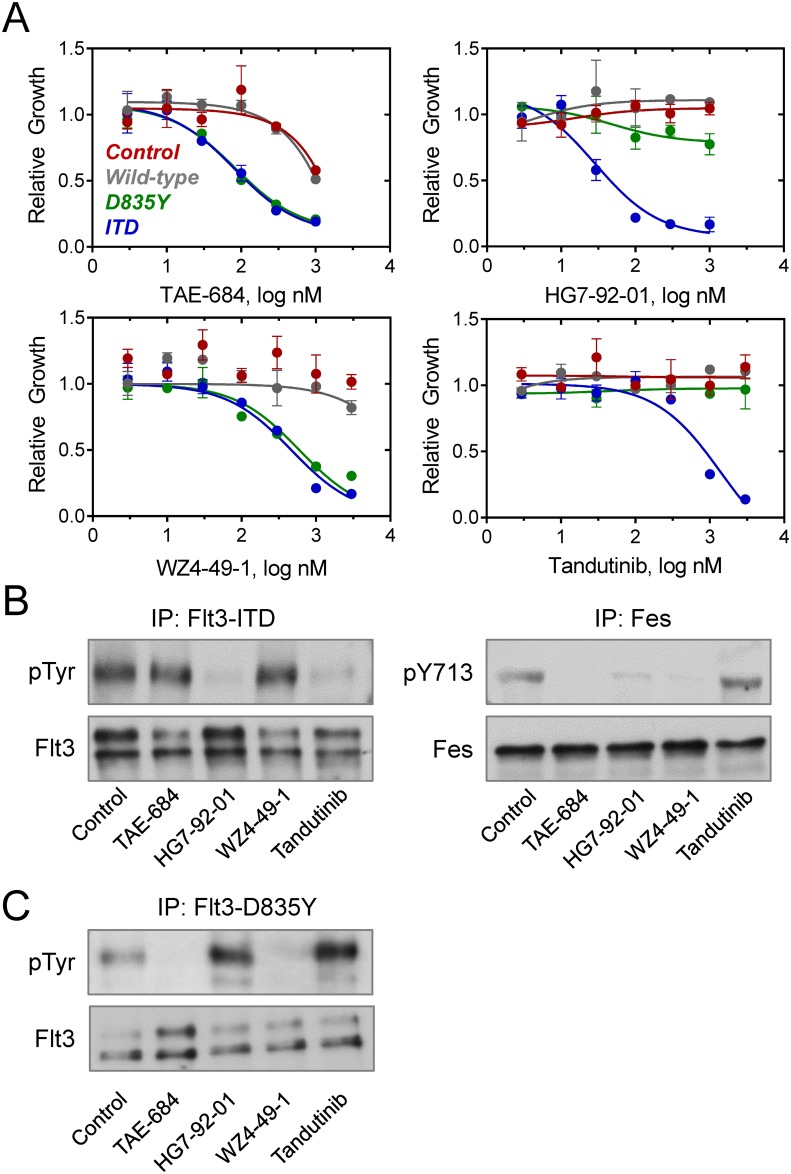
Inhibitor sensitivity of TF-1 cells transformed with wild-type (WT) and AML-associated mutants of Flt3. A) TF-1 cells expressing WT, ITD, and D835Y forms of Flt3, along with the empty vector control cells, were incubated with the kinase inhibitors shown over a range of concentrations and cell viability was measured 72 h later using the CellTiter-Blue Assay. Raw fluorescence data were corrected for background and then normalized to cultures grown in the presence of the DMSO carrier solvent. Each condition was performed in triplicate, and results are presented as the mean of the normalized values ± the SD. The resulting dose-response curves were fitted by non-linear regression analysis for estimation of the IC_50_ values (see Table 2). B) TF-1/Flt3-ITD cells were treated with TAE-684, HG7-92-01, and WZ4-49-1 at their calculated in vitro IC_90_ values for inhibition of Fes for 16 hours (or for Flt3-ITD in the case of tandutinib for 6 hours). Fes and Flt3 kinase proteins were then immunoprecipitated from cell lysates followed by immunoblotting with antibodies to phosphotyrosine (pTyr), the Fes activation loop phosphotyrosine (pY713), as well as the Flt3 and Fes proteins. This experiment was performed in triplicate with comparable results, and a representative example is shown. C) TF-1/Flt3-D835Y cells were treated with TAE-684, HG7-92-01, WZ4-49-1, and tandutinib as in part B, followed by immunoprecipitation of Flt3-D835Y and immunoblotting for Flt3 protein and phosphotyrosine content (pTyr).

We next investigated whether transformation of TF-1 cells with active forms of Flt3 resulted in sensitization to growth suppression by the panel of Fes inhibitors. Results obtained with TF-1 cells expressing Flt3-ITD recapitulated the effects seen in the Flt3-ITD^+^ AML cell lines. As shown in Fig 6A, all three compounds as well as tandutinib resulted in concentration-dependent inhibition of TF-1/Flt3-ITD cell growth. TAE-684 and HG7-92-01 were the most potent, with IC_50_ values of 82 and 29 nM, respectively (Table 2). The pyrazolopyrimidine Fes inhibitor WZ4-49-1, which displays selectivity for Fes vs. Flt3-ITD in vitro, inhibited TF-1/Flt3-ITD cell growth with an IC_50_ value of 466 nM. Tandutinib, on the other hand, was the weakest inhibitor of TF-1/Flt3-ITD cell growth, with an IC_50_ value of 1.4 μM, which may reflect its lack of inhibitory activity against Fes. Little to no growth inhibitory activity was observed with TF-1 cells expressing wild-type Flt3, or with the vector control cells, over the range of inhibitor concentrations tested although TAE-684 showed some nonspecific cytotoxicity in the low micromolar range.

**Table 2:** Sensitivity of TF-1 cells expressing wild-type, ITD and D835Y forms of Flt3 to inhibitors of Fes and Flt3 kinase activity. TF-1 cells were transduced with retroviruses expressing wild-type (WT), ITD or D835Y forms of Flt3, or an empty vector as negative control. Cells were grown in the presence of the kinase inhibitors shown, and IC_50_ values were calculated by non-linear curve fitting. The concentration-response curves used to generate these values are shown in Fig 6. Control cells in this experiment were TF-1 cells transduced with an empty retroviral vector.

Inhibitors		Inhibitor sensitivity of TF-1 Cell Populations (IC_50_, nM)			
Compound	Class	Control	Flt3-WT	Flt3-ITD	Flt3-D835Y
TAE-684	Diaminopyrimidine	> 1,000	> 1,000	82	83
HG7-92-01	Pyrrolopyridine	> 1,000	> 1,000	29	> 1,000
WZ4-49-1	Pyrazolopyrimidine	> 3,000	> 3,000	466	597
Tandutinib	Piperazinylquinazoline	> 3,000	> 3,000	1,404	> 3,000

To investigate whether each inhibitor was working through a mechanism in TF-1/Flt3-ITD cells similar to that observed in the Flt3-ITD^+^ AML cell lines, cells were treated with each compound at its calculated in vitro IC_90_ value for Fes (or Flt3-ITD for tandutinib). Flt3 and Fes were immunoprecipitated from treated cell lysates and immunoblotted for phosphotyrosine content as described above (Fig 6B). HG7-92-01 inhibited both Flt3-ITD and Fes, while WZ4-49-1 only inhibited the activity of Fes, consistent with the results in MV4-11 and MOLM-14 cells. TAE-684 also displayed identical results to those obtained with the Flt3-ITD^+^ AML cells, as it inhibited Fes but had no effect on Flt3-ITD, despite inhibitory activity against the Flt3-ITD kinase domain in vitro. Finally, tandutinib inhibited Flt3-ITD but not Fes, consistent with the in vitro kinase assay profile for this compound.

We also explored the sensitivity of TF-1 cells transformed with the Flt3 kinase domain point mutant D835Y, in which Fes activity is not enhanced. In these cells, TAE-684 treatment resulted in potent growth suppression, with an IC_50_ value of 83 nM (Fig 6A and Table 2). This observation suggests that the cellular activity of TAE-684 may result primarily from inhibition of the Flt3-D835Y mutant. HG7-92-01, on the other hand, was devoid of activity in TF-1/Flt3-D835Y cells, which is consistent with the reduced sensitivity of this compound to Flt3-D835Y in vitro in comparison to Flt3-ITD (Table 1). This observation, plus the finding that Flt3-D835Y is unable to activate Fes as a secondary inhibitor target, likely explains its lack of activity in TF-1/Flt3-D835Y cells. WZ4-49-1 inhibited TF-1/Flt3-D835Y cell growth with IC_50_ value of 597 nM. Interestingly, in vitro kinase assays show that the D835Y mutation sensitizes Flt3 to inhibition by this compound relative to Flt3-ITD, suggesting that it inhibits cell growth through direct inhibition of Flt3-D835Y in cells, since Fes is not active. Immunoblots shown in Fig 6C are consistent with these observations, where TAE-684 and WZ4-49-1 treatment resulted in complete inhibition of Flt3-D835Y in TF-1 cells, while HG7-92-01 was without effect. Similarly, tandutinib did not affect TF-1/Flt3-D835Y cell proliferation and had no inhibitory activity against Flt3-D835Y in the immunoblot assay, consistent with the reduced sensitivity of this Flt3 mutant to tandutinib in vitro (Table 1).

### HG7-92-01 induces apoptosis in patient-derived AML bone marrow cells but not in bone marrow cells from normal donors

**Fig 7:**
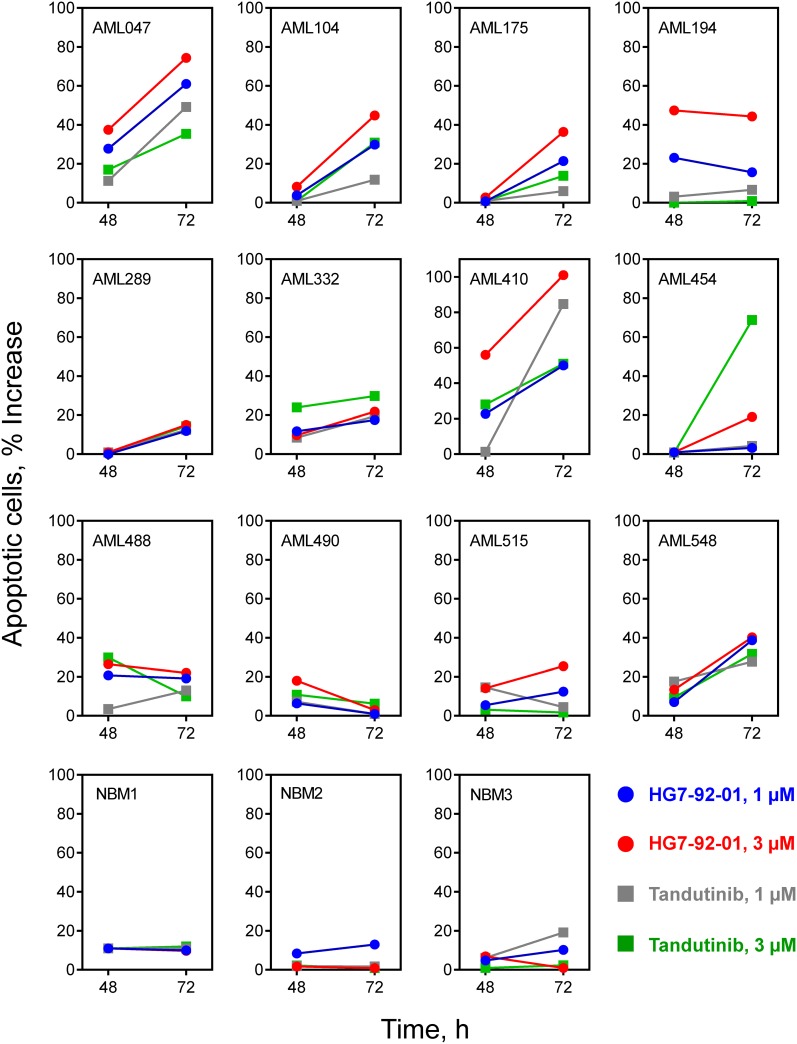
HG7-92-01 treatment induces apoptosis in patient-derived Flt3-ITD^+^ AML bone marrow cells. Cryopreserved biopsy samples from twelve randomly chosen Flt3-ITD^+^ AML patients (Table 3) were thawed and cultured on mitotically inactivated Hs27 feeder fibroblasts in the presence of myeloid cytokines as described in the text. After 24 h in culture, cells were treated with HG7-92-01 or tandutinib at 1 and 3 μM or with the DMSO carrier solvent as negative control. The presence of apoptotic cells was then assayed 48 and 72 h later by flow cytometry for cell-surface phosphatidylserine. Data are expressed as the increase in percentage of apoptotic cells observed at each time point relative to the DMSO control. To assess non-specific cytotoxicity, bone marrow mononuclear cells from three normal donors were cultured and treated with inhibitors under identical conditions, followed by flow cytometry for apoptotic cells. Characteristics of these three donors are as follows: normal bone marrow donor 1 (NBM1), female, 40 years; NBM2, male, 34 years; NBM3, male, 45 years.

In a final series of experiments, we investigated the most potent compound, HG7-92-01, for its ability to induce apoptosis in patient-derived AML bone marrow cells. For these studies, we obtained frozen bone marrow cells from twelve random Flt3-ITD^+^ patients (clinical characteristics are summarized in Table 3). These cells were cultured on feeder layers of mitotically inactive fibroblasts in the presence of the myeloid cytokines IL-3, IL-6 and SCF [17]. Twenty-four hours later, the cells were treated with either HG7-92-01 or tandutinib at final concentrations of 1 and 3 μM, or with DMSO as negative control. Cells from the treated cultures were then stained for apoptotic cells using antibodies to cell-surface phosphatidylserine [26], and analyzed by flow cytometry. As shown in Fig 7, HG7-92-01 treatment (3 μM) increased apoptotic responses by 30% or more in 6 out of the 12 samples examined (cases 047, 104, 175, 194, 410, and 548); in each case the fraction of apoptotic cells was higher in the HG7-92-01 treatment group compared to tandutinib group at the 3 μM concentration. Interestingly, most of the samples that did not respond to HG7-92-01 were also unresponsive to tandutinib (cases 289, 332, 488, 490, and 515), suggesting that additional cytogenetic changes or signaling pathways may be driving the survival of these AML cells. As a control for non-specific cytotoxicity, we also cultured bone marrow mononuclear cells from three normal donors under the same conditions, followed by inhibitor addition. Very little increase in apoptosis was observed in response to HG7-92-01 treatment of the normal cells (10% or less at 3 μM), demonstrating that its effects are specific for the responsive AML patient cell populations.

**Table 3:** Clinical characteristics of primary Flt3-ITD^+^ AML bone marrow samples. Age at diagnosis, gender, French-American-British (FAB) AML classification, and cytogenetic profile are shown for each patient; N/A, not available. Percent of leukemic blast cells present in peripheral blood (PB) and bone marrow (BM) are also shown. Samples are listed in order of ascending case number.

Patient	Age	Gender	FAB	Cytogenetics	% Blasts (PB)	% Blasts (BM)
047	47	F	M4	t(6,9)(p23;34)	14	59
104	66	F	M1	Normal	86	92
175	74	F	N/A	Normal	98	95
194	64	F	M5	t(6,11)(q27;q23)	20	74
289	70	M	N/A	Normal	62	70
332	46	M	M4	Normal	83	86
410	54	M	N/A	Normal	74	79
454	69	F	N/A	Complex	44	53
488	82	M	N/A	Normal	21	65
490	65	M	N/A	t(2;12)(p13;p13)	83	96
515	48	F	N/A	Normal	90	72
548	69	F	N/A	Normal	86	96

## Discussion

Previous studies have shown that the Fes tyrosine kinase is constitutively active in Flt3-ITD^+^ AML cells, and that RNAi-mediated knockdown of Fes expression causes growth arrest and apoptosis [14]. In the present study, we tested the efficacy of small molecule inhibitors of Fes kinase activity against Flt3-ITD^+^ AML cells. Our results show that direct inhibition of Fes kinase activity by a selective pyrazolopyrimidine compound (WZ4-49-1) resulted in growth suppression in three separate Flt3-ITD^+^ AML cell lines as well as human TF-1 myeloid cells transformed by retroviral transduction of Flt3-ITD. Furthermore, inhibitors with activity against both Fes and Flt3-ITD kinases in vitro blocked Flt3-ITD^+^ AML cell growth in the low to mid-nanomolar range, suggesting that dual inhibition of this key AML driver mutation and the proximal effector kinase Fes may provide added benefit in this type of AML.

Comparison of the inhibitor selectivity profiles against the Fes and Flt3-ITD kinases in vitro (Table 1) provides insight regarding the role of each kinase as an inhibitor target in TF-1/Flt3-ITD cells. For example, TAE-684 and HG7-92-01 both potently inhibited the growth of TF-1/Flt3-ITD cells, with IC_50_ values of 82 and 29 nM, respectively. These values are lower than the individual IC_50_ values obtained for either kinase in vitro (see Table 1), suggesting that dual inhibition of both Fes and Flt3-ITD may be responsible for their potent cellular growth inhibitory effects. On the other hand, WZ4-49-1 blocked TF-1/Flt3-ITD cell growth with IC_50_ values of 466 nM, a value in line with that observed for Fes in vitro (494 nM). In contrast, this compound is less potent against Flt3-ITD in vitro (1,390 nM). Taken together, these results imply that suppression of TF-1/Flt3-ITD cell growth by this pyrazolopyrimidine is primarily due to inhibition of Fes kinase activity. Finally, tandutinib inhibited TF-1/Flt3-ITD cell growth and Flt3-ITD kinase activity in vitro with similar potencies (1,404 vs. 788 nM, respectively), suggesting that tandutinib works primarily via Flt3-ITD. Note that tandutinib had no effect on Fes kinase activity in vitro, even at concentrations as high as 100 μM, nor did it affect Fes activation loop phosphorylation in tandutinib-treated TF-1/Flt3-ITD cells.

One unexpected discovery from our study was the observation that the AML-associated Flt3 kinase domain point mutant, D835Y, did not activate Fes in TF-1 cells transformed with this active form of Flt3. This observation is consistent with earlier work showing important differences in the downstream kinases and signaling pathways activated by Flt3-ITD vs. Flt3-D835Y in AML cells. For example, Src kinase is activated by Flt3-ITD but not by Flt3-D835Y in mouse 32D cells transformed with these active forms of Flt3 [27]. Furthermore, siRNA knockdown of Src inhibited Stat5 activity in these cells, suggesting Src links Flt3 to Stat5 activation downstream. Treatment of MV4-11 cells as well as AML patient samples with the FDA-approved CML drug dasatinib, which is also a very potent inhibitor of Src-family kinases, also reduced Stat5 activation in this study. Flt3-ITD signaling also suppresses the expression of two transcription factors essential for myeloid cell differentiation, while the D835Y does not [28]. These differences in signaling may help to explain increased disease severity observed in Flt3-ITD vs. Flt3-D835Y knock-in mouse models [29] and by extension the worse prognosis for patients with ITD vs. D835Y mutations in *FLT3*.

Consideration of differences in inhibitor potency between the different cell lines used in our study (Flt3-ITD^+^ AML cells such as MV4-11 vs. TF-1/Flt3-ITD cells) provides some additional mechanistic insight regarding inhibitor target kinase selection. For example, the potency of the pyrazolopyrimidine WZ4-49-1 was about two-fold higher in TF-1/Flt3-ITD cells compared to the Flt3-ITD^+^ AML cells. One possible explanation for this difference is that in MV4-11 and other Flt3-ITD^+^ AML cells, Flt3-ITD activates not only Fes, but also Syk [30] and several myeloid members of the Src kinase family (Hck, Lyn, and Fgr) [31–33]. Activation of these alternative kinase pathways may help to overcome the effect of Fes inhibition, resulting in reduced sensitivity to the inhibitor. Previous KINOMEscan data showed that a pyrazolopyrimidine inhibitor very similar in structure to WZ4-49-1 does not bind to Hck, Lyn or Syk [16], strongly suggesting that its growth suppressive activities in Flt3-ITD^+^ AML cell lines are primarily due to inhibition of Fes and not these other AML-associated kinases. On the other hand, TF-1/Flt3-ITD cells do not express Hck, Fgr, or Syk (data not shown), potentially making Flt3-ITD oncogenic signaling more dependent on Fes and thus more sensitive to this Fes-selective inhibitor.

While TAE-684 inhibited Flt3, Flt3-ITD, and Flt3-D835Y in vitro, treatment of Flt3-ITD-transformed cell lines with this compound did not affect Flt3-ITD phosphotyrosine content despite potent effects on cell growth. This apparent contradiction has been reported previously in a similar model system, in which mouse Ba/F3 cells were transformed to cytokine independence with Flt3-ITD [34]. In this case, treatment of Ba/F3 cells expressing Flt3-ITD with 100 nM TAE-684 had no effect on Flt3-ITD phosphotyrosine content. However, unlike our results, another report showed no effect of TAE-684 treatment on Flt3-ITD-transformed Ba/F3 cell proliferation [35]. Growth inhibition of Flt3-ITD-expressing cells by TAE-684 may therefore require inhibition of myeloid cytoplasmic kinases downstream of FLT3-ITD, including Fes, which are present in MV4-11 cells but may not be expressed in Ba/F3 cells because of their B-lymphoid origin. Previous KINOMEscan analysis shows that TAE-684 may also inhibit Syk and the myeloid Src-family members Hck, Lyn and Fyn in addition to Fes [16]. Multi-targeted activity against these AML-associated kinases may explain the potency of TAE-684 toward Flt3-ITD^+^ AML cell proliferation, despite its apparent lack of direct activity against the Flt3-ITD kinase in cells. Another interesting difference relates to the effect of TAE-684 on AML cells transformed with Flt3-ITD vs. the D835Y point mutant. While TAE-684 treatment did not affect Flt3-ITD phosphotyrosine content in Flt3-ITD^+^ AML cell lines or in TF-1 cells transformed with Flt3-ITD, TAE-684 completely suppressed Flt3-D835Y phosphorylation in TF-1 cells expressing this oncogene. This apparent difference in inhibitor sensitivity in the cellular environment further underscores what must be differences in kinase domain structure as well as association with Fes and other AML-related kinases as described above.

Our study also revealed an unexpected sensitizing effect of the Flt3-D835Y mutation towards the pyrazolopyrimidine inhibitor, WZ4-49-1. In vitro kinase assays showed that Flt3-D835Y was about 5-fold more sensitive to this compound compared to wild-type Flt3. This observation is consistent with the growth-suppressive action of WZ4-49-1 in TF-1/Flt3-D835Y cells, where this compound inhibits cell proliferation with an IC_50_ value that closely mirrors its potency against the Flt3-D835Y kinase domain in vitro. In addition, immunoblot data show complete inhibition of Flt3-D835Y tyrosine phosphorylation in TF-1/Flt3-D835Y cells treated with this inhibitor. In contrast, the D835Y mutation results in strong resistance to tandutinib and other Flt3 inhibitors in vitro, consistent with the lack of activity for this compound against TF-1/Flt3-D835Y cell proliferation.

In summary, results presented here provide the first evidence that ATP-site inhibitors with selectivity for Fes kinase activity cause growth arrest and induce apoptosis in Flt3-ITD^+^ AML cells. These results validate Fes as a target for drug development in Flt3-ITD^+^ AML, especially in cases where Fes is over-expressed and constitutively active. Furthermore, compounds with dual activity against both Fes and Flt3-ITD, such as HG7-92-01, induce apoptotic responses in FLT3-ITD^+^ AML cell lines and AML patient-derived bone marrow cells, suggesting that this therapeutic approach may be generally effective against a subset of Flt3-ITD^+^ AML cases. Future work will address this possibility using mouse xenograft models of AML.
